# Editorial Notes, News and Miscellany

**Published:** 1913-01

**Authors:** 


### Editorial Notes, News and Miscellany.

---

"The Red Back is doing a grand work, and Mrs. Daniel's department is superb."—Bogart.

---

Married, at Austin, Texas, December 10, Dr. L. B. Bibb, to Marion Alma Robinson, all of Austin.

---

The Regents of the University of Texas request of the Governor and the Legislature sufficient appropriations to bring the institution nearer to what the fathers of Texas demanded—"a University of the first class." The Journal earnestly supports the request and sees no reason why it should not be granted. The University now has in actual attendance more than 2000 students from 200 counties in Texas. Another thousand students are doing college work by correspondence, and still another thousand attend the summer school. Additional means is urgently needed. Send for the Press Bulletin—free.

---

Wolf.—Older members of the profession have heard the cry of "Wolf!" so often that they are a little suspicious of sure cures. They recall "gledestchine," "guiacol," "tuberculine," "606," etc. Dr. Friedmann's sure cure for tuberculosis is launched with a great flourish and it seems to be the real thing. Killing the germs is not curing the disease, however; the system is poisoned and the patient usually has septicemia and inanition, and months are required to restore the patient to health, if ever.

---

Dr. Preston, superintendent of the State Insane Asylum at Austin, in his annual report to the Governor, says that on account of the extremely crowded conditions, as many of the patients as possible have been kept on furlough. This is done to make room for acute cases, which accumulate very fast in the jails; that under this system insane resume their family relations and are apt, while on furlough, to transmit their unstable nervous organization to future generations, multiplying their kind, especially in cases of hereditary taint. He asks what can be done, however, when there is a continual demand for more room. Why, vasotomise them, of course.

---

CAPTAIN C. E. HAMPTON, U. S. A., retired, himself an author of note, says of Dr. Daniel's book, "The Strange Case of Dr. Bruno": "For scientific learning and sustained interest, it is one of the most remarkable productions of this age or of any other." (See advertisement.)

---

SALT LAKE CITY, Utah, December 25.—Sterilization of persons adjudged unfit to have offspring is advocated in the biennial report of the State Board of Insanity and the superintendent of the State Mental Hospital, filed with the Governor today. The following recommendations are made in the report:

Sterilization of all persons insane from hereditary cases.

Creation of a State board of eugenics to control marriages and issue certificates for licenses to only the fit.

An amendment to the penal code giving the upper court the power to impose sentence of sterilization in lieu of imprisonment in certain statutory crimes.

A law giving the board of pardons the power to make sterilization requirements precedent to parole or pardon from penal institutions, when in the judgment of the bureau of eugenics such is necessary.

---

TEXAS usually leads; Texas must follow in the matter of sterilizing the defectives. Dr. Daniel Parker, Representative from Robertson county, will present to the Legislature this month the same bill he had last session, amended so as to embrace the hereditary criminal and include the sterilization of women by the operation of "fallopiotomy." (That is a word I propose; there is no word for cutting the fallopian tubes.) And, by the bye, who first said "vasectomy"? It is incorrect; it should be vasotomy (to cut the vas); vasectomy means to excise, or cut out. (Get your dictionary.) But I digress. Dr. Parker's bill, drawn up by him and me in consultation with Judge C. H. Jenkins of the Court of Civil Appeals, provides that the superintendent of the asylum and one of his assistants shall decide which patients shall be operated on and call a surgeon to operate. The surgeon shall be paid by the State $3 for vasotomy and $5 for fallopiotomy. At the penitentiary, the superintendent (a layman) and the penitentiary physician shall decide. Every person discharged or furloughed from an asylum or paroled, discharged or pardoned from a penitentiary, unfit to be permitted to procreate, is to be sterilized. Texas was a pioneer in this proposed reform, but Indiana, Utah, Iowa, California, Connecticut, New Jersey and New York got ahead of us and have sterilization laws, and Texas must follow. This is our third attempt and will succeed.

